# Large Language Models Utility for Rapid On-Site Evaluation in Interventional Pulmonology

**DOI:** 10.3390/diagnostics16111658

**Published:** 2026-05-28

**Authors:** Maayan Flaschner, Mordechai Reuven Kramer, Alex Krolik, Evgeni Gershman, Anna Tovar, Dror Rosengarten, Shai Moshe Amor, Barak Pertzov, Moshe Heching, Osnat Shtraichman, Lev Freidkin

**Affiliations:** 1 Pulmonary Institute, Rabin Medical Center, Petah Tikva 4941492, Israel; 2 Gray School of Medicine, Faculty of Medical and Health Sciences, Tel Aviv University, Tel Aviv 6997801, Israel; 3 Department of Pathology, Rabin Medical Center, Petah Tikva 4941492, Israel; 4 Institute of Pulmonary Medicine, Tel Aviv Sourasky Medical Center, Tel Aviv 6423906, Israel

**Keywords:** rapid on-site evaluation, cytology, artificial intelligence, ChatGPT, Gemini, interventional pulmonology, large language model

## Abstract

**Background/Objectives**: Rapid on-site evaluation (ROSE) is a valuable technique in interventional procedures to immediately assess the adequacy and quality of biopsy specimens at the time they are obtained. The integration of artificial intelligence (AI) into ROSE workflows has demonstrated diagnostic accuracy comparable to that of experienced cytologists. However, clinical implementation of AI-based ROSE models is limited by complex and expensive development. In contrast, the use of free or near-free global large language models (LLMs) offers a significant advantage, making diagnostic support more accessible. Assess the diagnostic accuracy of the LLMs ChatGPT and Gemini in evaluating cytological smears during interventional pulmonology procedures. **Methods:** Retrospective evaluation of the efficacy of LLMs for assessment of cytological smears obtained from adult patients who underwent interventional bronchoscopic and ultrasound-guided biopsies between 2020 and 2025. Images of ROSE-prepared samples were analyzed by ChatGPT-4o, ChatGPT-5, ChatGPT-5 “thinking”, and Gemini 2.5 models. **Results:** Forty-eight procedures in 47 patients (mean age 65 years) were analyzed; 79% of biopsies were malignant. Using the final histopathology report as reference, cytologists achieved balanced accuracy of 0.75 (Gwet’s AC1 = 0.53, sensitivity 0.71, specificity 0.78). ChatGPT-5 “thinking” showed high concordance (accuracy 0.65, Gwet’s AC1 = 0.81, sensitivity 1.00, specificity 0.30). Gemini reached an accuracy of 0.59 (Gwet’s AC1 = 0.76, sensitivity 0.97, specificity 0.20). **Conclusions:** To our knowledge, this study is the first to evaluate LLM-assisted ROSE in interventional pulmonology. The results suggest the potential feasibility of integrating this AI technology into the workflow within the pulmonary division. Larger prospective studies are needed to confirm effects on diagnostic yield.

## 1. Introduction

Rapid on-site evaluation (ROSE) is a valuable technique in interventional procedures to immediately assess the adequacy and quality of biopsy specimens at the time of sampling. By confirming that diagnostically adequate material has been obtained, ROSE can increase diagnostic yield and improve overall procedural efficiency [1]. In the context of interventional pulmonology, ROSE has been particularly effective when used in conjunction with endobronchial ultrasound-guided transbronchial needle aspiration (EBUS-TBNA) and radial probe endobronchial ultrasound (r-EBUS) for diagnosing mediastinal lymph node and peripheral pulmonary lesions [2,3,4]. Unfortunately, recent surveys found a persistent shortage of cytologists despite a growing clinical demand for ROSE services [5,6,7].

The integration of artificial intelligence (AI) into ROSE workflows has demonstrated diagnostic accuracy comparable to that of experienced cytologists, with several models achieving over 90% sensitivity and specificity in identifying malignant cells [8,9,10,11,12,13,14]. A few different types of AI models have been used. Some rely on classical machine learning methods, such as the k-nearest neighbors algorithm (kNN), which classifies a new input by comparing it to similar previously labeled cases [14]. This method depends on predefined features and, therefore, is limited in its ability to recognize complex image patterns. More complex approaches utilize deep learning, particularly convolutional neural networks (CNNs), for image classification tasks [9,10,12]. These models can automatically extract complex morphological features from cytological images without the need for manual engineering. The last type of AI instruments for ROSE interpretation is an advanced segmentation network evolved from a fully convolutional network (FCN) that operates at the pixel level rather than producing a single classification output. This approach generates detailed maps of the image, identifying and localizing regions of interest, such as clusters of malignant cells [4,13].

These artificial intelligence systems enhance specimen adequacy assessment, reduce reliance on on-site cytologists, and show strong agreement with final histopathologic diagnoses. Despite their promising performance, such AI-based ROSE tools require complex development, large, annotated datasets, advanced computational infrastructure, and extensive validation, which can limit their scalability and clinical implementation.

In contrast, publicly available, free or near-free large language models (LLMs) such as ChatGPT [15] and Gemini [16] represent a different class of artificial intelligence. LLMs are big, general-purpose AI systems pretrained on massive heterogeneous data that can be adapted for a variety of specific tasks by focused instructions (a prompt) and a small amount of in-context data. These AI models offer a significant advantage by reducing development burden, enabling rapid deployment, and making advanced diagnostic support more accessible, particularly in low-resource clinical settings. In contrast to traditional large language models that were initially developed for language-related tasks, the new generation of LLMs is a multimodal system that combines language-processing and computer-vision capabilities. And therefore, although they are still commonly referred to as “language models,” current multimodal LLMs, in addition to text generation, can perform image-based analysis and have already been evaluated in radiology and dermatology [17,18].

In this study, we aimed to evaluate the diagnostic accuracy of large language AI models ChatGPT and Gemini for the assessment of cytological smears obtained during bronchoscopy and ultrasound-guided procedures and prepared on-site in a pulmonary institute, using histopathology as the reference standard.

## 2. Materials and Methods

The following section outlines the study design, procedure details, cytological image preparation, large language model prompting approach, and performance evaluation methods.

### 2.1. Study Design and Setting

We conducted a single-center, retrospective observational study on cytology smears obtained during interventional pulmonology procedures at a tertiary academic center (RMC—Rabin Medical Center, Israel). Standards for reporting diagnostic accuracy studies (STARD) and a targeted guideline for reporting large language models use (TRIPOD-LLM) checklists are available as Supplementary Materials Files S1 and S2.

### 2.2. Study Population

All patients who underwent bronchoscopy or US-guided biopsy of chest or neck lesions in the Pulmonary Institute at RMC between January 2020 and September 2025 were screened. The demographic, medical history, procedural and histopathological data were extracted from the institutional electronic medical record.

Inclusion criteria were adults (≥18 years) who underwent bronchoscopy or US-guided biopsy of a thoracic lesion at RMC during the study period, with at least one on-site prepared cytology smear. Cases where the cytology slide could not be reliably linked to the corresponding clinical or procedural record were excluded.

### 2.3. Procedures Equipment

For EBUS-TBNA procedures, we used a convex probe endobronchial ultrasound scope EBUS BF-UC180F with a ViziShot 2 22G needle and Evis EU-ME2 Ultrasound Processor (Olympus, Tokyo, Japan). Transbronchial cryobiopsy was performed with Erbecryo 2 station and flexible cryoprobes with 1.7 mm outer diameters (Erbe, Marietta, GA, USA). A US-guided chest/neck biopsy was obtained with a Temno Evolution 16G needle (Merit Medical, South Jordan, UT, USA) under TE7 portable ultrasound with C5-2s curved array (1.3–5.7 MHz; abdominal presets) and linear array (3.0 MHz–13.0 MHz; thyroid presets) transducers (Mindray, Shenzhen, China).

### 2.4. Slide Preparation and Image Acquisition

Cytology smears were prepared during the procedure according to guidelines [19] with immediate fixation and staining using Romanowsky DIFF-STAIN kit (Kaltek S.r.l., Padova, Italy). For each cytology smear, between one and three fields were selected for analysis by a pulmonologist experienced in weekly multidisciplinary meetings with the pathology department. The selected fields were focused on areas considered most diagnostically informative, based on cellular density and presence of atypical features. Digital high-resolution (3024 × 4032 pixels) images of each field were acquired using an upright Eclipse Ci-L light microscope (Nikon, Tokyo, Japan) at 40× objective magnification.

### 2.5. Cytological and Pathological Analysis

Cytology smears prepared during the procedures, along with tissue obtained from biopsy procedures, were sent for evaluation at the RMC Pathology Department. Cytological and histopathological evaluations were performed by a board-certified MD thoracic cytologist and pathologist, both with decades of experience in chest pathology. Final diagnoses were reviewed during a weekly multidisciplinary meeting, with discrepant cases undergoing additional review and verification. Finalized and verified diagnoses were included in the study dataset.

### 2.6. AI Evaluation Protocol

Each cytology image was evaluated independently by ChatGPT-4o, ChatGPT-5, ChatGPT-5 “thinking” (OpenAI) and Gemini 2.5 (Google) large language generative AI models. We developed a single, standardized prompt using published guidance on prompt engineering for academic and clinical tasks [20]. The four prompt key elements were: clear instruction, contextual constraints, input data, and an output format. In our case, the input defines the model’s role as a cytopathology consultant, limits it to provided reference sources for ROSE in lung and lymph node pathology [19,21,22], organizes the clinical and technical case information (type of interventional procedure, smear preparation and image acquisition techniques), and specifies a World Health Organization (WHO)-aligned structured response output format [19,21] (Supplementary Material File S3). The prompt was piloted on a small set of cytological images that represented both malignant and benign cells obtained from various procedures (two cases each of EBUS-TBNA, bronchial brushing, percutaneous US-guided true-core biopsy, and transbronchial cryobiopsy), which were not included in the main dataset of this research, and polished until it consistently produced interpretable, WHO-compatible responses.

We initially assessed diagnostic performance using all five WHO diagnostic categories for lung and lymph node cytopathology analysis (benign, atypical, suspicious for malignant, malignant, non-diagnostic). However, it became clear that several categories in our cohort contained too few cases to support reliable statistics across all five groups. For this reason, we switched to a dichotomous analysis, grouping together results showing “malignant” and “suspicious” categories into a single “non-benign” group versus “benign”. The “atypical” category was defined as a part of either the “non-benign” (strategy A) or “benign” (strategy B) group. This binary approach is not only better supported by the available sample size but also more closely reflects the key clinical decision point in the ROSE setting, as it will be discussed below.

### 2.7. Endpoints and Statistical Analysis

Descriptive statistics were used to summarize patient and procedure characteristics. Continuous variables are presented by mean and standard deviation, and categorical variables by number and percentage.

The primary endpoints were the diagnostic efficacy of the AI models for ROSE analysis, expressed as the Matthews correlation coefficient (MCC) and balanced accuracy for dichotomous classification (benign vs. non-benign). The second endpoints included comparative performance metrics of AI models and formal cytology, such as sensitivity, specificity, positive and negative predictive values (PPV and NPV), F1 score, and agreement statistics (Cohen’s κ and Gwet’s AC1). In addition, we estimated 95% confidence intervals for MCC and balanced accuracy using percentile bootstrap resampling (2000 iterations). McNemar’s exact test was used for paired comparisons; asymptotic Chi-square *p*-values with continuity correction are provided for completeness. In order to identify potential confounding factors and assess the robustness of LLM-assisted ROSE across different cytologic preparations, a specimen-type subgroup analysis was conducted. All analyses were performed in Python version 3.11.

### 2.8. Ethics and Privacy

All patient-level data were anonymized. Cytology images did not contain any direct identifiers; file names encoded only the study-specific patient and slide numbers. No identifiable demographic or clinical data were included in the AI prompts. The study was approved by the RMC Institutional Review Board (0493-25-RMC), and the patient’s informed consent was waived due to the retrospective design and anonymized data.

## 3. Results

### 3.1. Patient and Procedures Data

A total of 48 diagnostic procedures performed in 47 patients were included in this study. Of these, ROSE smears from 44 biopsies were evaluated by both a cytologist and a pathologist, whereas four cases (one EBUS-TBNA and three true-cut biopsies) were assessed by a pathologist only. Since the primary aim of our work was to evaluate the diagnostic performance of LLMs using the final histopathological diagnosis as the gold standard, these four biopsies were not excluded from the study cohort. Baseline patient characteristics are summarized in Table 1. The mean patient age was 65.4 ± 10.5 years, and 26 patients (55.3%) were men. Smoking status was available for 46 patients; of these, 28 (60.9%) were current or former smokers.

The most common indication for the biopsy was evaluation of a solitary space-occupying pulmonary lesion, accounting for 26 (54.2%) cases followed by isolated mediastinal lymphadenopathy without a discrete pulmonary lesion (seven procedures, 14.6%), supraclavicular and cervical lesions (seven biopsies, 14.6%), lung infiltration with or without mediastinal involvement (four cases, 8.3%), endobronchial (three procedures, 6.3%) and chest wall lesions (onr biopsy, 2.1%).

Most samples were obtained by bronchial brushing (24 procedures, 50.0%), followed by endobronchial ultrasound-guided transbronchial needle aspiration (EBUS-TBNA; 12, 25.0%), percutaneous ultrasound-guided true-core biopsy (11, 22.9%), and transbronchial cryobiopsy in one procedure (2.1%). Procedural details are shown in Table 1.

The final histopathological diagnosis was malignant in 38 (79.2%) biopsies and included non-small cell lung cancer (14 cases, 33.3%), neuroendocrine tumors (eight cases, 16.7%), metastases from other organs (nine cases, 18.8%), lymphoma (two cases, 4.2%), and other neoplastic diseases in five cases (10.4%). Benign pathology was identified in 10 samples (20.8%). A detailed histopathological description is presented in Table 2.

### 3.2. Diagnostic Performance of Cytology and AI Models

**Figure 1:**
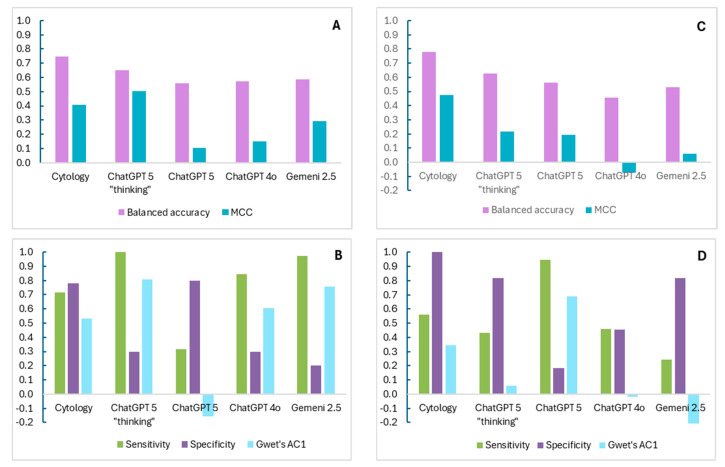
Graphical comparison of agreement and discrimination for cytology and generative AI models for two dichotomization strategies: including “atypia” in “non-benign” (**A**,**B**) or “benign” (**C**,**D**) category. (**A**,**C**) Primary endpoints, (**B**,**D**) agreement and classification performance, AC1—first-order agreement coefficient; MCC—Matthews correlation coefficient.

Matthews correlation coefficient (MCC) between formal cytology and histopathology report was 0.41 (95% CI, 0.13–0.66) and 0.47 (95% CI, 0.31–0.64) with a balanced accuracy of 0.75 (95% CI, 0.57–0.89) and 0.78 (0.70–0.86) for dichotomization strategies A and B, respectively. ChatGPT models showed varying levels of concordance with pathology as the highest observed for 5 “thinking” model for strategy A group with MCC 0.5 (95% 0.0–0.76) and balanced accuracy 0.65 (95%, CI 0.5–0.81) outperforming Gemini 2.5 that achieved MCC 0.29 (95% CI, −0.09–0.62) and balanced accuracy 0.59 (95% CI, 0.48–0.74) (Table 3, Figure 1A,C).

For strategy A, most AI models favored sensitivity over specificity. In particular, ChatGPT-5 “thinking” correctly identified all non-benign cases, with a sensitivity of 1.00, PPV of 0.84 and NPV of 1.00, but at the cost of a lower specificity of 0.30. Gemini performed similarly, with a sensitivity of 0.97, specificity of 0.20, PPV of 0.82 and NPV of 0.67 (Table 4, Figure 1B). Compared to AI models, with a disproportion between sensitivity and specificity, cytologist reports have a more balanced diagnostic profile, with moderate sensitivity and higher specificity (0.71 and 0.78, respectively). Analysis according to dichotomization with “atypia” results as part of a “benign” group (strategy B) demonstrated a shift to higher specificity at the expense of sensitivity in most modalities. For example, cytology achieved a specificity of 1.00 and a sensitivity of 0.56. The opposite pattern was demonstrated for the ChatGPT-5 model, which showed high sensitivity (0.95) with low specificity (0.18). These differences between the two dichotomization strategies were also reflected in Gwet’s AC1 and F1 scores (Table 4, Figure 1D).

Analysis with Gwet’s AC1 agreement coefficient, which adjusts for chance agreement and is more stable than Cohen’s kappa in imbalanced datasets, indicating moderate to substantial agreement of cytologists and most LLMs with histopathology despite the high prevalence of malignant samples. For dichotomization strategy A, ChatGPT-5 “thinking” and Gemini 2.5 showed the highest agreement (Gwet’s AC1 of 0.81 and 0.76, respectively), whereas conventional cytology demonstrated moderate agreement (0.53). In contrast, ChatGPT-5 had a negative Gwet’s AC1 values (−0.16), meaning agreement was worse than expected by chance. Under strategy B, most LLMs showed low agreement (0.06 for ChatGPT-5 “thinking” and −0.25 for Gemini), while it was high for ChatGPT-5 (0.69). Cytology remained relatively stable across both classification frameworks (0.53 vs. 0.35) (Table 4).

Paired comparison of AI models and cytologists demonstrated a statistically significant difference in classification patterns between human expert, ChatGPT-5 “thinking” (*p* = 0.001) and Gemini 2.5 (*p* < 0.001) for dichotomization strategy A. Under strategy B, the asymmetry observed for ChatGPT-5 “thinking” was no longer statistically significant (*p* = 0.383), suggesting that a substantial proportion of disagreement in strategy A was related to interpretations of atypical results (Table 5).

Given that ROSE is inherently a cytological assessment, an additional analysis of LLM diagnostic performance using cytological report as the final reference was conducted (Supplementary Material File S4). These results show MCC values near zero or negative (−0.14 for Gemini 2.5 and −0.03 for ChatGPT-5 “thinking”) and indicate that AI models’ results do not align well with cytological assessments.

A subgroup analysis of diagnostic performance across various modalities was performed to evaluate the impact of specimen type on diagnostic accuracy (Table 6). Overall, the results suggest that the effect of specimen type was not uniform across models. For instance, under strategy A, the balanced accuracy of cytology and ChatGPT-5 was higher in EBUS samples than in brushing (0.75 for both vs. 0.69 and 0.5, respectively). The opposite, although to a lesser extent, was demonstrated for ChatGPT-5 “thinking” and Gemini 2.5 (0.63 vs. 0.67 and 0.56 vs. 0.36, respectively). The true-cut subgroup was limited by the absence of true negative cases, precluding meaningful estimation of specificity and agreement metrics.

## 4. Discussion

In this study, we evaluated the feasibility of using large language AI models as part of rapid on-site evaluation during various interventional pulmonology procedures. To the best of our knowledge, this is the first study to investigate the use of an LLM for this purpose.

Previous studies demonstrating high effectiveness of artificial intelligence for ROSE in bronchoscopy have relied on classical machine learning algorithms or deep neural network-based image analysis models, which are well-suited to cytological evaluation but are more expensive, less accessible, and require longer development and validation [8,9,14]. Despite encouraging results of AI instruments for ROSE interpretation, several challenges remain. Most studies to date are single-center and retrospective, limiting external validity, and are primarily focused on binary classification (malignant vs. benign), with limited capability for detailed tumor subtyping or integration of clinical context. Ethical considerations, such as algorithmic bias and the risk of overreliance on AI systems, also require careful attention before widespread implementation in clinical work.

In recent years, following their release for free and open public access, large language models have come to widespread use across many domains of our lives [23,24,25,26]. In medicine, their availability, low cost, and efficiency have made them practical tools within clinical workflows with a growing demand for integration into therapy decision pathways to improve patient experience and enhance diagnostic capabilities [17,18,25,27,28].

We evaluated Google’s Gemini 2.5 and three configurations of the OpenAI platform—ChatGPT-4o, ChatGPT-5, and the ChatGPT-5 “thinking” models. In our cohort, ChatGPT-4o and ChatGPT-5 showed consistently poor diagnostic behavior (low MCC and moderate accuracy) and less stable agreement with the histopathological reference standard (low to moderate Gwet’s AC1). In contrast, the best-performing model, ChatGPT-5 “thinking” demonstrated better performance with high sensitivity and Gwet’s AC1, but with lower accuracy than an experienced cytologist. While sensitivity in our study (up to 100%) can be compared with results achieved by AI deep learning convolutional neural networks (ranging from 96.1% for the CNN model [12] to 97.4% for a kNN model [14]), direct comparison of other performance parameters is more challenging. This is largely because previous studies have reported heterogeneous metrics, often focusing on conventional (“regular”) accuracy (reported values range from 84.6% accuracy for CNN models [9] to 98.3% for the modified FCN model [13]) rather than balanced accuracy that is more reliable in medical datasets (see below).

The findings of our work suggest that with a carefully constructed prompt, an LLM-based assistant may contribute to improving diagnostic yield in interventional pulmonology by reducing the risk of false-negative ROSE interpretations.

However, it is important to interpret these results within the context of our dataset, which was small and dominated by malignant diagnoses. In such an imbalanced cohort, both conventional accuracy and Cohen’s kappa may be misleading, whereas Matthews correlation coefficient, balanced accuracy, and Gwet’s AC1 were chosen for more cautious assessment. MCC measures the strength of correlation between predicted and true classifications, ranges from −1 for inverse prediction to +1 for perfect prediction, remains reliable and does not inflate performance under one class domination. Balanced accuracy ranges from 0 (worse than random classification) to 1 (perfect discrimination), gives equal weight to both “benign” and “non-benign” cases and provides a fair assessment of performance in an uneven dataset. Gwen’s first-order agreement coefficient measures agreement between raters while adjusting for chance agreement. It ranges from −1 for systematic disagreement to +1 for perfect agreement, is much less sensitive to prevalence inequality and better than Cohen’s κ, which reflects true diagnostic concordance as it avoids the κ paradox—where high observed agreement may paradoxically yield low κ values in datasets dominated by a single outcome category. Nevertheless, even after implementing statistical metrics suitable for our dataset, there is a discrepancy between moderate accuracy and a strong level of agreement with the final pathology results that reflects the complexity of retrospective diagnostic tool evaluation rather than calling into question the model’s clinical potential. Future prospective studies are needed to validate LLMs performance on real-world clinical data.

Notably, in this study, we used AI models via their standard user interface, without application programming interface (API) access. API-based use would allow control of model version and parameters, automated processing of multiple cases and greater reproducibility. However, its disadvantages are additional costs, the need for dedicated software development and secure integration with clinical systems, which is critical to ensure patient data privacy and confidentiality. Given the exploratory nature of this study and the relatively small cohort, we opted for a structured prompt approach with constrained reference material derived from purchased digital cytopathology textbooks [19,21]. Each case was entered individually using standardized clinical descriptors without inclusion of expanded clinical data, such as imaging findings, underlying comorbidities, sex, or patients’ age (Supplementary Material File S3), allowing us to assess the initial feasibility of LLM-assisted ROSE interpretation under controlled conditions. One possible direction for future work could involve shifting from a standardized fixed prompt to context engineering that incorporates non-identifiable patient-specific information, enabling a more personalized approach [29].

The main limitations of our study are a small cohort, a retrospective design, and the combination of high sensitivity and low specificity, reflecting a substantial proportion of false-positive results. One explanation for this pattern is selection bias: given that ROSE is typically requested when clinical suspicion of malignancy is high, our dataset thus consisted of mostly malignant samples, whereas the number of benign smears was small (38 vs. 10). Although we addressed it by choosing a reliable statistical endpoint, a larger prospective study is needed to reassure results.

Another explanation of low specificity relates to the categorization of cytologic findings by language models. The models were constrained to assign one of five cytologic categories: malignant, suspicious, atypical, benign, or non-diagnostic. For the purposes of diagnostic analysis and due to a small cohort size, results were divided into two groups: “benign” and “non-benign”. Whereas under strategy A, any malignant, suspicious for malignancy and atypical interpretations were considered as a “positive” result when compared with a malignant histopathological diagnosis, strategy B defined atypical interpretations as benign (“negative”). We believe that both these approaches can reflect the clinical role of ROSE, which in some procedures aims to provide a definitive tissue diagnosis, while in certain clinical settings (low-grade malignancy, time-sensitive procedure in unstable patient, etc.), it rather guides real-time sampling of the target lesion. However, under the dichotomization strategy A, cases classified by the LLM as “atypical” but with final benign histopathology—such as granulomas or chronic inflammation—were labeled as non-concordant and counted as false positives, despite representing clinically reasonable correlation. This limitation was partially addressed in strategy B, in which atypical interpretations were classified within the benign/negative category.

The choice of dichotomization strategy influenced the diagnostic behavior of both cytology and LLMs. Under strategy A, the best-performing AI models demonstrated very high sensitivity but poor specificity (ChatGPT-5 “thinking” with 1.0 and 0.3, Gemini 2.5 with 0.97 and 0.2 for sensitivity and specificity, respectively). When atypical results were reclassified in strategy B, the sensitivity of ChatGPT-5 “thinking” and Gemini 2.5 decreased substantially (to 0.43 and 0.24, respectively), while specificity increased (up to 0.82 for both models). Similar trends were observed in additional performance and classification metrics such as F1 score, MCC, and Gwet’s AC1. These findings suggest that a substantial proportion of false-positive results under strategy A originated from the interpretation of atypical results. At the same time, despite shifts in sensitivity and specificity, LLMs had a relatively stable or only moderately changed balanced accuracy between strategies. Interestingly, the ChatGPT-5 model behaved in the opposite direction, increasing sensitivity from 0.32 to 0.95 and lowering specificity from 0.8 to 0.18 under strategies A and B, respectively. In contrast to AI models, conventional cytology demonstrated a more balanced diagnostic profile and was less affected by the change in dichotomization strategy compared with LLM’s that may reflect the ability of experienced cytologists to distinguish uncertain atypia from malignant cytomorphology. Paired comparison analysis under strategy A showed systematic directional disagreement of ChatGPT-5 “thinking” and Gemini 2.5 with cytology. After moving atypia from “non-benign” to “benign” group, this asymmetry attenuated for ChatGPT-5 “thinking” and disappeared for ChatGPT-4o, suggesting that atypical interpretations represented a major source of discordance between cytology and certain LLMs. Google’s Gemini 2.5 model remained significantly different from cytology under both strategies, indicating persistent systematic differences in classification behavior beyond atypical cases alone. Overall, these results demonstrate that the classification of “borderline” cytologic categories, such as “atypia”, has a significant effect on the diagnostic performance of AI models in ROSE.

Importantly, the relatively low rate of pre-procedural PET-CT imaging in our cohort (29.2%) may have contributed to more empiric lesion targeting and increased prevalence of diagnostically ambiguous or borderline tissue samples, which affects the likelihood of intermediate ROSE interpretations. At present, an optimal analytic strategy to overcome this intermediate category limitation is not clear. One potential approach is to incorporate specimen adequacy, which may better reflect the clinical utility of LLM-assisted ROSE and warrants further investigation.

In addition, diagnostic performance varied across specimen types, suggesting that intrinsic sample characteristics may influence model behavior. For example, blood-rich and morphologically complex samples, such as brushing (Supplementary Material File S4 and Figure 1A), potentially contribute to increased wrong interpretations, whereas more structured cytologic material, as seen in EBUS-TBNA samples (Supplementary Material File S4 and Figure 1B), may facilitate more reliable diagnosis.

Moreover, like larger studies involving complex AI models [10,12,14], our work shares the inherent limitation that the images of cytology smear fields were selected by clinicians and focused on areas judged to be most diagnostically informative. While this approach reflects routine clinical practice, it may introduce field-of-view selection bias. The models, therefore, evaluated limited fields chosen by humans rather than performing whole-slide scanning of independently self-detected areas. The possible solution to avoid this limitation is using the whole-slide imaging technique that allows more comprehensive and objective analysis, but demands high-end hardware, massive data storage, and is time-intensive [9,11].

Although the high-sensitivity LLM results are encouraging, false-positive interpretations may still influence procedural strategy, leading to premature cessation of procedure and reduced diagnostic yield. It must be emphasized that AI output should not be used as a definitive source, but rather as a supportive tool for clinical judgment. Anticipating cases where AI assessment might differ from clinician impression, we suggest developing a workflow protocol that clearly defines decision hierarchies and needs to be validated prospectively to ensure that AI enhances rather than complicates ROSE efficiency and patient safety.

## 5. Conclusions

In summary, this retrospective study is, to our knowledge, the first to assess the use of LLM technology as part of ROSE for the evaluation of thoracic lesions sampled during interventional pulmonology procedures. Although limited by a small sample size, we demonstrated preliminary results regarding the feasibility of integrating this AI technology into the routine workflow within the pulmonary institute. In the setting of rising demand for on-site biopsy evaluation and a shortage of cytologists and resources, the use of freely available LLMs may offer a practical, scalable tool to support existing diagnostic workflows. Our findings suggest that LLM-assisted ROSE could function as a real-time supportive tool, particularly by reducing the risk of false-negative interpretations and assisting in decisions regarding continued sampling when on-site cytology expertise is limited or unavailable. Given their high sensitivity, these models may help identify cases that warrant additional tissue acquisition, thereby potentially improving overall diagnostic yield. However, in our opinion, the clinical use of such systems should remain restricted, carefully supervised, and limited to an adjunctive role within the diagnostic workflow rather than serving as an independent decision-making modality. Larger prospective studies are needed to validate these findings and to clarify the impact of LLM integration on diagnostic yield.

We anticipate that using future generations of AI models with API access will allow us to mitigate the limitations resulting from modest cohort sizes and to handle intermediate or indeterminate cytological categories with greater diagnostic precision.

## Figures and Tables

**Table 1:** Demographics and baseline characteristics (47 patients and 48 procedures).

Patient Characteristics (*n* = 47)	
Age (years), mean ± SD	65.4 ± 10.5
Sex, *n* (%)	
Men	26 (55.3)
Women	21 (44.7)
Smoking history ^1^, *n* (%)	
Current or former smoker	28 (60.9)
Non-smoker	18 (39.1)
Procedure characteristics (*n* = 48)	
Indication, *n* (%)	
SOL	26 (54.1)
without mediastinal lymphadenopathy	17
with mediastinal lymphadenopathy	9
Isolated mediastinal lymphadenopathy	7 (14.6)
Supraclavicular and cervical lesions	7 (14.6)
solitary lesion	3
with systemic lymphadenopathy	4
Lung infiltration with or without mediastinal involvement	4 (8.3)
Endobronchial lesion	3 (6.3)
Chest wall lesion	1 (2.1)
Biopsy modality, *n* (%)	
Bronchial brushing	24 (50.0)
EBUS-TBNA	12 (25.0)
Percutaneous US-guided true-core biopsy	11 (22.9)
Transbronchial cryobiopsy	1 (2.1)
Pre-procedural PET-CT, *n* (%)	
Performed	14 (29.2)
Not performed	34 (70.8)

^1^ Based on data available for 46 patients. EBUS-TBNA—ultrasound-guided transbronchial needle aspiration; PET-CT—combined positron emission tomography and computed tomography scans; SOL—space-occupying lesion; US—ultrasound.

**Table 2:** Pathological diagnosis from 48 procedures.

Diagnosis	Biopsy, *n* (%)
Benign (*n* = 10)	
Normal lung	4 (8.3)
Sarcoid-like granulomas	3 (6.3)
Organizing pneumonia	2 (4.2)
IgG4-related disease	1 (2.1)
Malignant (*n* = 38)	
NSCLC	14 (33.3)
NET	8 (16.7)
SCLC	6
Neuroendocrine carcinoma	2
Metastases	9 (18.8)
Melanoma	3
Carcinoma of unknown origin	3
Metastatic gland duct carcinoma	1
Adenocarcinoma enteric pattern	1
Neurogenic spindle cell lesion	1
Lymphoma	2 (4.2)
Others	5 (10.4)
Poorly differentiated carcinoma	2
Plasmacytoma	1
Anaplastic epithelial carcinoma	1
Leiomyosarcoma	1

IgG—immunoglobulin G; NET—neuroendocrine tumor; NSCLC—non-small cell lung cancer; SCLC—small cell lung cancer.

**Table 3:** Primary overall diagnostic performance metrics of cytology and generative AI models compared with histopathology in dichotomic benign/non-benign result analysis. Results are presented for two dichotomization strategies: including “atypia” in the “non-benign” (A) or “benign” (B) category.

	Strategy A		Strategy B	
	MCC (95% CI)	Balanced Accuracy (95% CI)	MCC (95% CI)	Balanced Accuracy (95% CI)
Cytologist	0.41 (0.13–0.66)	0.75 (0.57–0.89)	0.47 (0.31–0.64)	0.78 (0.70–0.86)
ChatGPT-5 “thinking”	0.50 (0.0–0.76)	0.65 (0.5–0.81)	0.22 (−0.06–0.43)	0.63 (0.47–0.76)
ChatGPT-5	0.10 (−0.18–0.32)	0.56 (0.4–0.69)	0.19 (−0.13–0.54)	0.56 (0.46–0.71)
ChatGPT-4o	0.15 (−0.17–0.46)	0.57 (0.41–0.73)	−0.07 (−0.35–0.21)	0.46 (0.29–0.63)
Gemini 2.5	0.29 (−0.09–0.62)	0.59 (0.48–0.74)	0.06 (−0.23–0.30)	0.53 (0.38–0.66)

CI—confidence intervals; MCC—Matthews correlation coefficient.

**Table 4:** Secondary classification and agreement performance metrics of cytology and generative AI models compared with histopathology in dichotomic benign/non-benign result analysis.

	Sensitivity	Specificity	PPV	NPV	Cohen’s Kappa	Gwet’s AC1	F1 Score	TP	TN	FP	FN
Dichotomization strategy A											
Cytologist	0.71	0.78	0.93	0.41	0.37	0.53	0.81	25	7	2	10
ChatGPT-5 “thinking”	1.00	0.30	0.84	1.00	0.40	0.81	0.92	38	3	7	0
ChatGPT-5	0.32	0.80	0.86	0.24	0.06	−0.16	0.46	37	2	8	1
ChatGPT-4o	0.84	0.30	0.82	0.33	0.15	0.6	0.83	32	3	7	6
Gemini 2.5	0.97	0.20	0.82	0.67	0.23	0.76	0.89	12	8	2	26
Dichotomization strategy B											
Cytologist	0.56	1.00	1.00	0.40	0.37	0.35	0.72	19	10	0	15
ChatGPT-5 “thinking”	0.43	0.82	0.89	0.30	0.16	0.06	0.58	16	9	2	21
ChatGPT-5	0.95	0.18	0.80	0.50	0.16	0.69	0.86	35	2	9	2
ChatGPT-4o	0.46	0.45	0.74	0.20	−0.06	−0.02	0.57	17	5	6	20
Gemini 2.5	0.24	0.82	0.82	0.24	0.03	−0.25	0.38	9	9	2	28

AC1—first-order agreement coefficient; FN—false negative; FP—false positive; TN—true negative; TP—true positive; NPV—negative predictive value; PPV—positive predictive value.

**Table 5:** Paired comparison between cytology and generative AI models in result analysis was performed using McNemar’s test.

	Strategy A		Strategy B	
	*p*-Value	Chi-Square *p*-Value	*p*-Value	Chi-Square *p*-Value
ChatGPT-5 “thinking” vs. cytology	0.0013	0.0022	0.3833	0.3827
ChatGPT-5 vs. cytology	0.0768	0.0801	0.0639	0.0662
ChatGPT-4o vs. cytology	0.0059	0.0071	1	1
Gemini 2.5 vs. cytology	0.0005	0.0012	0	0

**Table 6:** Performance difference across diagnostic modalities according to specimen type.

	Sensitivity		Specificity		MCC		Balanced Accuracy	
	EBUS	Brush	EBUS	Brush	EBUS	Brush	EBUS	Brush
Dichotomization strategy A								
Cytologist	0.50	0.72	1.00	0.67	0.46	0.35	0.75	0.69
					(0.0–0.83)	(−0.07–0.71)	(0.57–0.93)	(0.44–0.9)
ChatGPT-5 “thinking”	1.00	1.00	0.25	0.33	0.43	0.52	0.63	0.67
					(0.0–0.84)	(0.0–0.85)	(0.5–1.0)	(0.50–0.88)
ChatGPT-5	1.00	0.00	0.50	1.00	0.63	0	0.75	0.5
					(0.0–1.0)	(0.0–0.0)	(0.5–1.0)	(0.5–0.5)
ChatGPT-4o	0.88	0.78	0.25	0.33	0.16	0.11	0.56	0.56
					(−0.32–0.71)	(−0.26–0.57)	(0.36–0.9)	(0.36–0.8)
Gemini 2.5	0.88	1.00	0.25	0.17	0.16	0.36	0.56	0.36
					(−0.32–0.71)	(0.0–0.69)	(0.36–0.9)	(0.5–0.8)
Dichotomization strategy B								
Cytologist	0.25	0.65	1.00	1.00	0.29	0.59	0.63	0.82
					(0.0–0.56)	(0.38–0.84)	(0.5–0.79)	(0.71–0.93)
ChatGPT-5 “thinking”	0.75	0.00	0.50	1.00	0.25	0.00	0.63	0.50
					(−0.32–0.82)	(0.0–0.0)	(0.33–0.94)	(0.50–0.50)
ChatGPT-5	0.75	0.00	0.50	1.00	0.25	0.00	0.63	0.50
					(−0.32–0.82)	(0.0–0.0)	(0.33–0.94)	(0.50–0.50)
ChatGPT-4o	0.00	0.47	0.50	0.43	−0.63	−0.09	0.25	0.45
					(−1.0–0.0)	(−0.49–0.33)	(0.0–0.50)	(0.22–0.69)
Gemini 2.5	0.88	1.00	0.25	0.14	0.16	0.32	0.56	0.57
					(−0.32–0.71)	(0.0–0.65)	(0.36–0.90)	(0.50–0.75)

EBUS—endobronchial ultrasound-guided transbronchial needle aspiration; MCC—Matthews correlation coefficient.

## Data Availability

The raw data supporting the conclusions of this article will be made available by the authors on request and Institutional Review Board approval.

## Supplementary Materials

The following supporting information can be downloaded at: https://www.mdpi.com/article/10.3390/diagnostics16111658/s1, Supplementary Material File S1: STARD checklist, Supplementary Material File S2: TRIPOD-LLM checklist, Supplementary Material File S3: Example of the prompt for ChatGPT for ROSE evaluation of lymph node biopsy obtained by EBUS-TBNA, Supplementary Material File S4: Diagnostic performance of LLM models using cytological diagnosis as the final reference and representative cytological smears images from different biopsy modalities.

## Abbreviations

The following abbreviations are used in this manuscript:

AI	artificial intelligence
AC1	first-order agreement coefficient
API	application programming interface
CI	confidence intervals
CNN	convolutional neural networks
CT	computed tomography
EBUS	endobronchial ultrasound
FCN	fully convolutional network
IgG	immunoglobulin G
kNN	k-nearest neighbors algorithm
LLM	large language model
MMC	Matthews correlation coefficient
NET	neuroendocrine tumor
NPV	negative predictive value
NSCLC	non-small cell lung cancer
PET	positron emission tomography
PPV	positive predictive value
ROSE	rapid on-site evaluation
SCLC	small cell lung cancer
SOL	space-occupying lesion
TBNA	transbronchial needle aspiration
US	ultrasound
