# Supplementary material for: Large Language Models Utility for Rapid On-Site Evaluation in Interventional Pulmonology
Source: Diagnostics (Basel). 2026 May 28;16(11):1658. doi: 10.3390/diagnostics16111658 (PMC13257399; doi:10.3390/diagnostics16111658)
Supplement: Supplementary file 1 [file diagnostics-16-01658-s001.zip › supplemental material File S4.pdf]

Supplement File S4

Table S2. Diagnostic performance of LLM models using cytological diagnosis as the final reference (based on 44 biopsies with available cytology results).

	MCC (95% CI)	Balanced Accuracy (95% CI)	Sensitivity	Specificity	PPV	NPV	Cohen's Kappa	Gwet's AC1
**Dichotomization strategy A**								
**ChatGPT-5 "thinking"**	-0.03 (-0.27–0.25)	0.49 (0.42–0.57)	0.93	0.06	0.61	0.33	-0.02	0.37
**ChatGPT-5**	0.16 (-0.17–0.42)	0.54 (0.46–0.64)	0.96	0.12	0.63	0.67	0.10	0.44
**ChatGPT-4o**	0.18 (-0.15–0.47)	0.57 (0.44–0.71)	0.85	0.29	0.66	0.56	0.16	0.38
**Gemini 2.5**	-0.14 (-0.43–0.16)	0.43 (0.30–0.57)	0.22	0.65	0.50	0.34	-0.11	-0.21
**Dichotomization strategy B**								
**ChatGPT-5 "thinking"**	0.00 (-0.30–0.30)	0.50 (0.36–0.64)	0.32	0.68	0.43	0.57	0.00	0.10
**ChatGPT-5**	0.12 (-0.19–0.33)	0.53 (0.45–0.62)	0.95	0.12	0.45	0.75	0.06	0.06
**ChatGPT-4o**	0.22 (-0.08–0.51)	0.61 (0.46–0.75)	0.58	0.64	0.55	0.67	0.22	0.24
**Gemini 2.5**	-0.21 (-0.45–0.07)	0.41 (0.31–0.53)	0.11	0.72	0.22	0.51	-0.19	0.04

AC1 - first-order agreement coefficient; CI - confidence intervals; FN – false negative; FP – false positive; MCC - Matthews correlation coefficient; NPV – negative predictive value; PPV – positive predictive value; TN – true negative; TP – true positive

Figure S1. Representative cytological smears from brushing and EBUS-TBNA illustrating differences in background composition. (A) Bronchial brushing specimen demonstrating a blood-rich background. (B) EBUS-TBNA smear showing comparatively less hemorrhagic background, with some debris and preparation-related artifacts.

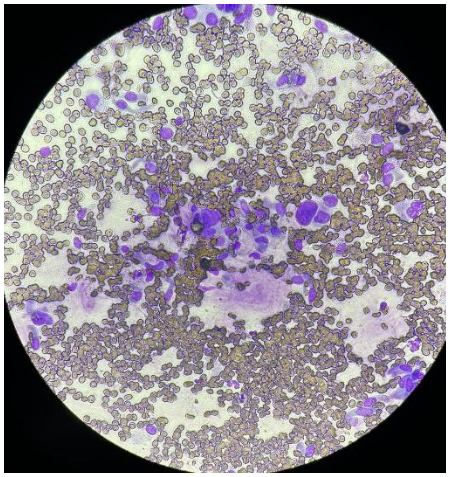

A

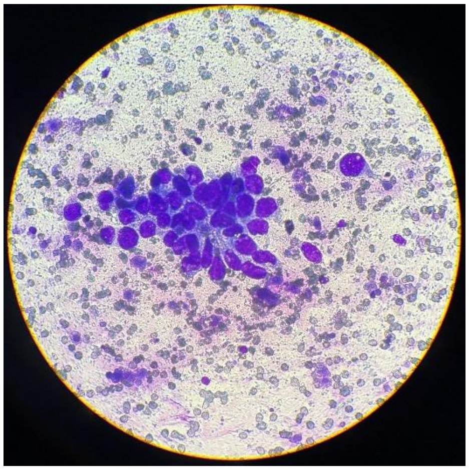

B
